# Assessment of Salivary Adipokines Resistin, Visfatin, and Ghrelin as Type 2 Diabetes Mellitus Biomarkers

**DOI:** 10.1155/2018/7463796

**Published:** 2018-02-01

**Authors:** Mythily Srinivasan, Melinda L. Meadows, Lisa Maxwell

**Affiliations:** ^1^Departments of Oral Pathology, Medicine and Radiology, Indiana University School of Dentistry, Indianapolis, IN, USA; ^2^Preventive and Community Dentistry, Indiana University School of Dentistry, Indianapolis, IN, USA

## Abstract

Type 2 diabetes mellitus (T2DM) is emerging as a metabolic epidemic worldwide. Pathologically, dysregulation of many biological pathways precedes hyperglycemia and the clinical diagnosis of T2DM. Changing trajectories along the process of T2DM development necessitates frequent measurement of biomarkers for early identification of at-risk individuals and successful prevention. Increase in circulating inflammatory adipokines has been suggested as predictive of T2DM. Human saliva is an easily accessible biospecimen amenable for painless frequent collection and possesses nearly 50% of serum proteome. In this study, we measured the adipokines resistin, visfatin, TNF-*α*, and ghrelin as markers for T2DM in unstimulated whole saliva (UWS) using specific assay kits. Resistin and visfatin concentrations were significantly higher in T2DM saliva. Although the concentration of acylated or unacylated ghrelin was lower in diabetic saliva, the decrease was not significant. Since resistin and visfatin are biomarkers integral to T2DM pathology, their salivary assessments may receive clinical acceptance.

## 1. Introduction

The World Health Organization estimated that globally 422 million adults were living with diabetes in 2014 [1]. Type 2 diabetes mellitus (adult-onset/noninsulin-dependent diabetes: T2DM) accounts for 90–95% of all diabetes [2]. The disease develops insidiously through periods of increased insulin secretion, insulin resistance, impaired glucose tolerance, and *β*-cell dysfunction [3]. Consistently, the most acceptable markers for T2DM diagnosis are based on measurements of blood glucose and glycosylated hemoglobin c (HbA1c), an indicator of average glycemic control [4]. However, research elucidating the disease pathogenesis suggests that multiple mechanisms including chronic inflammation, obesity, lipotoxicity, and oxidative stress contribute to the glucose dysregulation in T2DM [5, 6]. Hence, several hypothesis-based nonglycemic biomarkers have been assessed as risk factors for diabetes [7, 8].

Adipokines are polypeptides secreted by adipocytes, inflammatory cells, and other cells. They regulate multiple physiological functions including energy balance, insulin sensitization, appetite regulation, and inflammatory response [9]. It has been suggested that activation of the adipokine resistin in the islet cells of the pancreas inhibits cell surface glucose transporters and thereby insulin signaling [10, 11]. Visfatin, also known as pre-B-cell colony-enhancing factor (PBEF), has been described as an adipokine with a potential glucose-lowering effect due to its nicotinamide phosphoribosyltransferase (NAMPT) activity [10, 12, 13]. Ghrelin, originally identified as a growth hormone secretagogue with orexigenic and lipogenic effects, has also been shown to play significant roles in glucose regulation. While acylated ghrelin has been shown to exert hyperglycemic effects leading to insulin resistance, the unacylated ghrelin counters hyperglycemia and enhances insulin sensitivity [14, 15]. Biomarker studies showed that the circulating levels of resistin and visfatin are upregulated in T2DM [13, 16–18]. On the other hand, the plasma concentration of acylated ghrelin has been shown to be lower in T2DM individuals as well as in their healthy offspring [19–21].

Since monitoring of serological parameters typically involves invasive techniques with associated pain and distress, efforts are directed at identifying noninvasive measures for frequent monitoring of diabetes. Some of the alternative methods evaluated include assessing skin autofluorescence for accumulation of advanced glycation end-products and measuring analytes in exhaled breath, urine, or saliva [5, 22–24]. Human saliva is a rich reservoir of analytes consisting of over 3652 proteins and 12,562 peptides and shares nearly 51% of proteins and 79% of peptides with the serum proteome and peptidome, respectively [25, 26]. Alterations in the salivary flow and composition in diabetes are well documented [27, 28]. Both glucose and immunoreactive insulin are increased in saliva and are correlated with plasma levels in T2DM patients [29–32]. Circulating biomolecules are thought to reach saliva by either active (e.g., sIgA) or passive transportation (e.g., steroids) or ultrafiltration (e.g., creatinine) or from crevicular fluid [26, 33]. The objective of this study is to compare the salivary levels of two proinflammatory adipokines, namely, resistin and visfatin, and that of the anti-inflammatory adipokine ghrelin between healthy and T2DM individuals.

## 2. Materials and Methods

### 2.1. Study Population

All participants were recruited from patients attending the Indiana University School of Dentistry after obtaining informed consent in full accordance with the Indiana University Institutional Review Board. The study population consisted of twenty periodontally healthy individuals with self-reported T2DM and HbA1c values. Twenty gender-matched individuals with no known oral or systemic condition were recruited as control group. It was estimated that this sample size will be sufficient at 80% power to detect a difference in means of 0.91, assuming a common standard deviation of 1 using a two group *t*-test with a two-sided significance level of 0.05.

### 2.2. Collection and Processing of Unstimulated Whole Saliva (UWS)

UWS was collected at approximately the same time of day by the drooling method as described [34, 35]. Briefly, subjects were asked to refrain from eating or drinking for 2 h prior to saliva collection. At least 2 mL of UWS was collected by passively drooling into a chilled centrifuge tube for 5–10 min. The tubes were codified and transferred on ice to the laboratory for processing. Each sample was clarified by centrifuging at 3500 rpm at 4°C for 10 min and stored in Complete™ Protease Inhibitor Cocktail (Roche, Mannheim, Germany). The supernatant-clarified saliva was stored at −80°C until further analysis.

### 2.3. Enzyme-Linked Immunosorbent Assay (ELISA) for Resistin and Visfatin

All UWS samples were depleted of amylase and immunoglobulins by incubating serially with antihuman amylase mAb (1 : 2500, cat. no. ab8944; Abcam) and protein G beads (Miltenyi Biotec Inc.) at 4°C. Total protein of the precleaned saliva samples was determined by spectrophotometry and ranged between 1.3 and 7.7 mg/mL [36]. Volume equal to 1 µg of total protein in precleaned UWS was assessed for resistin, visfatin, and ghrelin per using specific sandwich ELISA kits (item no. 10007610 81, part no. 579020-96, and part no. 10006306-96, resp.; Bertin Pharma/Cayman Chemical, Ann Arbor, MI, USA) following manufacturer's instructions. The cytokines TNF-*α* and IL-6 in saliva were measured using specific ELISA kits (BD Biosciences, CA, USA) following the manufacturer's instructions.

### 2.4. Statistical Analysis

For all biomarkers, statistical significance between the healthy and diabetes cohorts was determined by two-tailed paired *t*-tests; *p* < 0.05 was considered significant.

## 3. Results

### 3.1. Clinical Characteristics

The study cohort consisted of twenty individuals with self-reported T2DM and twenty healthy individuals (Table 1). The average age of T2DM cohort was 56.5 yrs and that of healthy group was 48 yrs. The average HbA1c value of the T2DM group was 5.4 ± 1.9%. Since the HbA1c values reported were measured within the past three months, the range is consistent with the diagnostic criteria for T2DM [37].

### 3.2. Salivary Cytokines in T2DM

**Figure 1:**
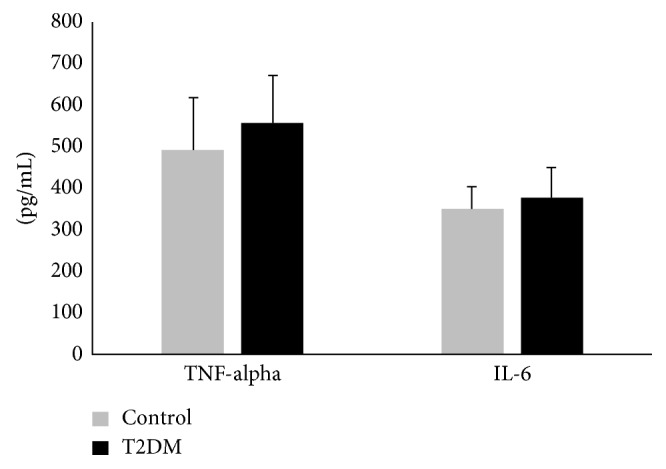
Salivary TNF-*α* and IL-6 in T2DM: Unstimulated whole saliva (UWS) was collected from 20 T2DM individuals and 20 healthy individuals. Each UWS sample was depleted of amylase and immunoglobulins. Precleaned UWS was assessed for TNF-*α* and IL-6 using specific sandwich ELISA kits.

We observed that the UWS concentration of IL-6 and TNF-*α* was not significantly different between T2DM and healthy individuals with no periodontitis (Figure 1). Similar observation of comparable salivary IL-6 levels between systemically healthy and diabetes individuals with healthy periodontium has been reported by others [38].

### 3.3. Differential Expressions of Visfatin, Resistin, and Ghrelin in Diabetic Saliva

**Figure 2:**
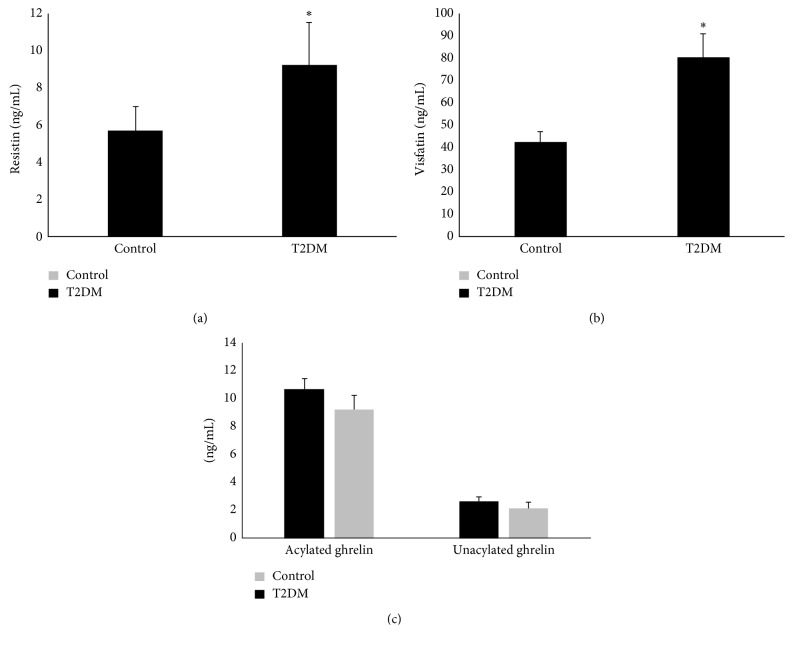
Salivary resistin, visfatin, and ghrelin in T2DM: Unstimulated whole saliva (UWS) was collected from 20 T2DM individuals and 20 healthy individuals. Each UWS sample was depleted of amylase and immunoglobulins. Precleaned UWS was assessed for (a) resistin, (b) visfatin, and (c) acylated and unacylated ghrelin using specific assay kits. ^∗^*p*<0.05.

We observed that the UWS from T2DM subjects possessed significantly elevated levels of resistin (9.2 ± 2.3 ng/mL) and visfatin (80.2 ± 42.3 ng/mL) as compared to that from control subjects (5.7 ± 1.3 ng/mL and 46.0 ± 17.5 ng/mL, resp.) (Figures 2(a) and 2(b)). No significant difference was observed in the salivary concentration of either unacylated (9.2 ± 4.3 ng/mL and 10.7 ± 5.6 ng/mL, resp.) or acylated (2.7 ± 2.3 ng/mL and 2.2 ± 1.4 ng/mL, resp.) ghrelin between the T2DM and the control group (Figure 2(c)).

## 4. Discussion

Escalating global burden of T2DM underscores the need for multipronged screening strategies for early identification of individuals at high risk [7, 39]. Furthermore, elucidation of the molecular pathogenesis has shown that the processes that lead to T2DM are initiated very early with a long lag phase between the disease onset and the clinical diagnosis [6]. Many cross-sectional studies have evaluated multiple serum proteins as predictive biomarkers for T2DM [5, 7, 39].

Potential applications of salivary biomarkers for T2DM have gained importance with the establishment of shared characteristics of salivary and serum proteomes [26]. It has been suggested that the increased basement membrane permeability often associated with diabetes is a potential mechanism for the increased passage of proteins and metabolites from the exocrine glands as well as for the enhanced leakage of serum-derived components into whole saliva [26, 33, 40].

Clinical application of salivary components as potential biomarkers is likely to be better accepted for molecules that correlate with the pathological process of T2DM. Considerable evidence suggests that the T2DM is a multifactorial disease involving dysregulation of various biological pathways such as inflammation, adipokine signaling, and incretin signaling [6]. The prodiabetic effects of the adipokine resistin have been attributed to inhibition of insulin signaling and a pro-inflammatory mechanism that culminates in *β*-cell loss [9, 10, 13, 41]. The adipokine visfatin has been shown to exhibit glucose-lowering and insulin-mimicking/insulin-sensitizing effects [9, 10, 12, 13]. Circulating levels of both resistin and visfatin are upregulated in T2DM [12, 13, 18, 42]. Previously, others have shown positive correlation between the serum and salivary levels of these two adipokines [42–44]. Here, we observed that the salivary resistin and visfatin concentrations are significantly elevated in T2DM. Similar observations of elevated salivary visfatin and resistin have been reported earlier in chronic periodontitis and diabetes [42, 43, 45].

Ghrelin, the orexigenic peptide hormone also affects glucose metabolism. Circulating levels of ghrelin rise before and fall after a meal, thereby contributing to appetite and weight gain [15]. Plasma concentration of ghrelin has been negatively correlated with insulin resistance [14, 46]. We observed that the salivary concentration of both acylated and unacylated ghrelin was lower in T2DM saliva than that in healthy saliva although the difference did not reach statistical significance. Others have reported significant reductions in acylated ghrelin in diabetic saliva [47]. The difference may be attributed to the time and method of sample collection and preparation and the method of ghrelin assessment.

## 5. Conclusions

Population-based long-term studies suggest that the biomarker trajectories along the course of T2DM development diverge over time [48, 49]. This suggests that repeated measures of mechanism-based biomarkers will increase the predictive value of diabetes risk scores. The noninvasive nature and the feasibility of frequent sampling for real-time monitoring are significant advantages of saliva over peripheral blood as specimen for diagnostic/prognostic applications. In addition to the practical benefits of eliminating the need for a phlebotomist, reduced transmission of infectious disease by eliminating needle sticks and greater ease of testing of special populations of patients (e.g., institutional and children) make the assessment of biomarkers in human saliva an attractive economic strategy [24, 50]. Elevated salivary resistin and visfatin in saliva that have also been shown to correlate with serum levels suggest that the two adipokines could represent potential noninvasive T2DM biomarkers [41, 43, 44]. However, caution must be exercised since the type of sample (stimulated/unstimulated; whole/glandular), circadian variations, and susceptibility to preprocessing as well as oral health/disease are some of the confounding parameters that should be addressed in the biomarker interpretations and implementation [51, 52].

## Figures and Tables

**Table 1:** Demographic characteristics, HbA1c, and total salivary protein content.

		Healthy	T2DM
Number of individuals	M	10	12
	F	10	8
Age (yrs)		48 (range: 42–55)	56.5 (range: 45–58)
% A1C			7.85
			Range: 6–12
Salivary protein (mg/mL)		3.4 ± 1.6	5.4 ± 1.9
